# The Influence of Weekly Training Load on Match Physical Demands in Spanish Professional Soccer Players: A Full In-Season Study

**DOI:** 10.3390/s25082413

**Published:** 2025-04-11

**Authors:** José C. Ponce-Bordón, Jorge Polo-Tejada, David Lobo-Triviño, Borja Sanabria-Pino, Javier Raya-González, Alberto Muñoz, Tomás García-Calvo

**Affiliations:** 1 Faculty of Sport Sciences, University of Extremadura, 10003 Caceres, Spain; joponceb@unex.es (J.C.P.-B.); jpolotej99@gmail.com (J.P.-T.); bsanabriapino@gmail.com (B.S.-P.); tgarciac@unex.es (T.G.-C.); 2 Research Group on Sport and Physical Education for Personal and Social Development, Faculty of Education Sciences and Psychology, University of Cordoba, 14071 Cordoba, Spain; rayagonzalezjavier@gmail.com; 3 Sport Performance Area, CP Cacereño, 10003 Caceres, Spain; ammpf1@gmail.com

**Keywords:** external load, monitoring, football, tracking technology, training

## Abstract

**Highlights:**

**What are the main findings?**

- Trivial-to-small correlations were observed between the weekly training load (TL) and the match physical demands of that week for different external load variables.
- During MD–3, the greatest values of total distance (TD), medium-speed running (MSR), very high speed running (VHSR), Sprint distance, high-speed running (HSR), and player load (PL) occurred, and during MD–4, the highest values of accelerations (ACC) and decelerations (DEC) occurred, showing that the hardest training sessions were those days.

**What is the implication of the main finding?**

- A correct weekly TL distribution could optimize the subsequent performance of soccer players.
- Practitioners should reduce the volume of the training sessions after heavy training sessions characterized by high external load in MD–4 and MD–3.

**Abstract:**

This study aimed (i) to analyze the relationship between weekly accumulated training load (TL) and match physical demands in the same week and (ii) to describe the training/match ratios of different external load measures considering variations across different training days. Twenty-one Spanish male professional soccer players were involved in the study. Total distance (TD), medium speed running (MSR, distance 10.8–18.0 km·h^−1^), high-speed running (HSR, >21 km·h^−1^), very high-speed running (VHSR, 18.0–25.2 km·h^−1^), sprinting-speed running distance (Sprint, >25.2 km·h^−1^), player load (PL), number of accelerations (ACC), and decelerations (DEC) were recorded during training sessions and matches using 10 Hz GPS devices. Correlations between the weekly TL and match physical demands were trivial and negative for TD (*r* = −0.08) and PL (*r* = −0.05); trivial and positive for MSR (*r* = 0.02), HSR (*r* = 0.07), Sprint (*r* = 0.09), and DEC (*r* = 0.06); and small and positive for VHSR (*r* = 0.22) and ACC (*r* = 0.19). The greatest TD, MSR, VHSR, Sprint, HSR, and PL values and their derivate ratios occurred in MD–3. The present study highlights the need for soccer athletes to be exposed to similar demands to those observed during matches.

## 1. Introduction

Soccer is a team sport that is highly demanding in terms of total distance (TD), high-intensity running distances, and accelerations (ACC) and decelerations (DEC) [1]. In recent years, an evolution in match physical demands during soccer matches has been observed, with players covering greater distances at higher speeds and increasing the number of high-intensity actions [2,3,4,5,6]. Therefore, match analysis is always a hot topic of study to adapt players’ training periodization to competitions’ demands [7]. In this vein, strength and conditioning coaches aim to understand data from match analysis to prepare players for competition, seeking to improve their performance and achieve sport success [8], while reducing the injury risk in football [9].

The authorization granted by FIFA for the use of GPS during competitions has allowed for significant progress in football research, linking data from match performance to training sessions aimed at injury risk reduction [10]. This method can improve the quality of the different sessions conducted by football players [11], both in professional and non-professional soccer teams [12]. So, the advancement of new technologies has enabled a more detailed understanding of training load (TL) [13], allowing for the quantification of the workload on a microcycle-by-microcycle basis and during training sessions and matches [14].

Despite the importance of knowing match physical demands and weekly TL, there is a lack of studies analyzing the relationships between weekly TL and match physical demands. In this regard, some studies have been focused on the weekly TL distribution [15], examining its relationship with sport success [16,17]. Other studies have investigated the effects of TL variation across different weeks of the season [18]. Some authors have explored the percentage of workload in relation to competition during training [19,20], but few studies have looked at how this weekly workload affects match performance [21], and even fewer have analyzed its effect over an entire season, considering several external load variables. The increase in knowledge related to the optimum relationship between weekly TL and match physical demands could be useful for provide precise stimuli by considering the needs of each player [22]. Weekly TL could serve as readiness for matches and providing a form of injury risk prevention and an opportunity to develop fitness levels [23].

Another of the main benefits of using GPS is understanding the TL in relation to the session objectives and the microcycle day (e.g., MD–4, strength), as well as the work performed each day [24,25]. This concept is well-known as relative TL or training/match ratios (TMr; [20]). The assessment of TMr is useful to determine what actions and physical demands are replicated during training sessions compared to matches or even assess the weekly TL of the entire mesocycle [26]. For example, in a soccer team from the Dutch first division league, TD and high-intensity runs were significantly lower than those performed during competition; although, ACC and DEC were more similar to those in matches [27]. This method applies individual loads from training and represents an important procedure for adjusting individual weekly TL, according to the match physical demands [16].

To address the gaps observed in the scientific literature regarding to this topic, a full season of a professional male soccer team was monitored using GPS devices. Specifically, the aim of this study was: (i) to analyze the relationship between weekly accumulated TL and match running performance in the same week and (ii) to describe the training/match ratios of different external load measures considering variations across different training days. Based on previous studies, we hypothesized that an increase in weekly TL leads to greater match physical demands. That is, weekly TL variables were positively correlated to match physical demands.

## 2. Materials and Methods

### 2.1. Study Design

A retrospective, descriptive, and longitudinal study was conducted with a professional soccer male team to examine the relationship between weekly TL and match physical demands during the same week. All matches and training sessions were monitored throughout the 2023/24 season (from 3 September 2023 to 5 May 2024, i.e., just the competitive phase of the season). Only weeks with one official match and with four or more training sessions were included in this study. This decision was made to reduce the variability among comparisons [20]. Thus, only 34 weeks’ worth of data and a total of 171 training sessions related to the in-season period were included in the analysis. Data were recorded daily (i.e., training and matches) to analyze differences in external load variables. Based on the proximity to the match and the team’s training schedule, each training day was categorized as follows [24]: MD+1, the day following the match (*n* = 447 observations); MD–4, four days before the match (*n* = 576 observations); MD–3, three days before the match (*n* = 537 observations); MD–2, two days before the match (*n* = 505 observations); MD–1, one day before the match (*n* = 460 observations); and MD, match day (*n* = 548 observations).

### 2.2. Participants

Twenty-one professional male soccer players (age = 25.7 ± 2.7 years old; height = 180.7 ± 7.3 cm; weight = 75.4 ± 6.9 kg) voluntarily accepted to participate in this study. All players belonged to the same team, which competed in the Second Federation championship (Fourth Spanish Division) during the 2023/24 season. Goalkeepers participated in the training sessions but were excluded from the analysis due to their specific roles. During the experimental period, players completed five training sessions per week, each lasting an average of 110 min, and played one official match per week. Thus, 3073 total match and training observations were included. Participants were fully informed about the objectives and potential risks of the study, and they provided written informed consent prior to participation. This study was conducted in compliance with the Declaration of Helsinki (2013) and received ethical approval from the University of Extremadura’s Ethics Committee (protocol number: 33/2024).

### 2.3. Instruments and Variables

To measure the external workload during both training sessions and matches, GPS devices (Vector S7, Catapult Sports, Melbourne, Australia) were utilized. Each player wore the same device throughout the season, positioned in the center of the upper back. The Vector S7 features a tri-axial accelerometer, gyroscope, and magnetometer, each with a sampling rate of 100 Hz, while the GPS operates at 10 Hz [28]. Following the manufacturer’s guidelines, the devices were activated 15 min prior to matches or training sessions to ensure proper satellite signal acquisition and synchronization. Previous research has shown that similar Catapult devices demonstrate high reliability [29,30]. External workload demands were assessed using the following variables: total distance (TD) covered (in meters); distance covered at 10.8–18.0 km·h^−1^ (medium-speed running, MSR); distance covered above 18.0 km·h^−1^ (high-speed running, HSR), distance covered at 18.0–25.2 km·h^−1^ (very high-speed running, VHSR); distance covered above 25.2 km·h^−1^ (sprinting-speed running distance, Sprint); accelerations above 4 m·s^−2^ (ACC, n°); decelerations beyond −4 m·s^−2^ (DEC, n°); and the accelerometer-derived player load (PL, in meters), which includes vertical, anterior–posterior, and medial–lateral axes. All variables are reported as mean values per session.

### 2.4. Training and Match External Load Ratios Explanation

The mean of each external load variable during all training sessions and matches of the week was calculated for players who competed in the match and who had participated in all training sessions during the week of the match. In addition, the sum of each variable during all training sessions of the week (excluding official matches) was also calculated per player, thus providing the weekly load for each variable. Then, a ratio dividing the daily external load by the match physical demands of the same week was calculated for the players. The training/match ratio (TMr) was calculated for each player (individualized) based on the following formula: TMr = daily external load/match physical demands [20]. Thus, a TMr was calculated for each external load variable. Therefore, the following TMr values were obtained: (i) TDr (total distance ratio); (ii) MSRr (medium-speed running ratio); (iii) VHSRr (very high-speed running ratio); (iv) Sprintr (sprinting-speed running ratio); (v) HSRr (high-speed running ratio); (vi) ACCr (accelerations [>4 m·s^−2^] ratio); (vii) DECr (decelerations [>4 m·s^−2^] ratio); (viii) PLr (player load ratio). In addition, match physical demands (excluding warm-up) of players in the same week were also recorded for all players who participated in matches at least 80 min from the same week.

### 2.5. Statistical Analysis

Data analysis was conducted using RStudio (version 2024.12.0+467) and the *dplyr*, *tidyr*, and *psych* packages for data manipulation and statistical calculations. Firstly, linear mixed models (LMM) were used to analyze the effects of training day on external workload variables using the *lme4* package [31]. A hierarchical level structure with the soccer player as the nesting unit of the training and match observations was considered for the analysis. Therefore, a two-level hierarchy was modelled for the analysis. The external load variables (TD, MSR, HSR, VHSR, Sprint, ACC, DEC, and PL) were included as dependent variables in the models, and training and match day (MD+1, MD–4, MD–3, MD–2, MD–1, MD) were the independent variables included as fixed effects. The variable soccer player was considered as the random effect in the analysis. Values were represented as coefficients and standard error (Coeff ± SE). Then, a general multilevel modelling strategy was applied where fixed, and random effects in different steps were included [32,33]. Additionally, the total weekly external load was calculated by the sum of daily averages to obtain a weekly total load for each variable.

Secondly, to examine the relationship between the weekly accumulated external load and match values for each variable, a correlation analysis was performed on the calculated weekly sums of external load variables excluding matches. The Pearson’s correlation coefficient (*r*) was applied to quantify the strength and direction of the linear relationships between variables. Missing data were handled using pairwise deletion to maximize the use of available data for each pair of variables. In addition, a correlation matrix was visualized through a heatmap, employing a gradient color scale ranging from blue (positive correlation) to red (negative correlation), with white indicating no correlation. The magnitudes of the correlations were interpreted based on the following thresholds: [0.0–0.1] = trivial; [0.1–0.3] = small; [0.3–0.5] = moderate; [0.5–0.7] = large; [0.7–0.9] = very large; [0.9, 1.0] = nearly perfect [34]. Confidence intervals of 95% were used for the correlation values (*r*). Key R packages included were *tidyverse* (for data manipulation and preparation), *corrplot* (to compute and visualize correlation matrices), and *ggplot2* (for advanced and customizable heatmap visualization). Finally, to evaluate the relative external load during training sessions compared to matches, the descriptive statistics of daily TMr were calculated depending on training day by repeated-measure ANOVA.

## 3. Results

### 3.1. Daily and Match External Load According to Training Day

Table 1 shows the descriptive statistics (coefficients ± standard error) of daily and match external load measures according to the type of day. During MD, the soccer players performed the highest values of all external load variables included in the analysis. Excluding matches, the greatest TD, MSR, VHSR, Sprint, HSR, and PL occurred in MD–3. However, the highest ACC and DEC occurred in MD–4. Contrarily, the lowest values of TD, VHSR, HSR, ACC, DEC, and PL appeared in MD–2. During MD–1, the soccer players performed the lowest values of MSR and Sprint.

### 3.2. Correlations Between Weekly External Load and Match Physical Demands

**Figure 1:**
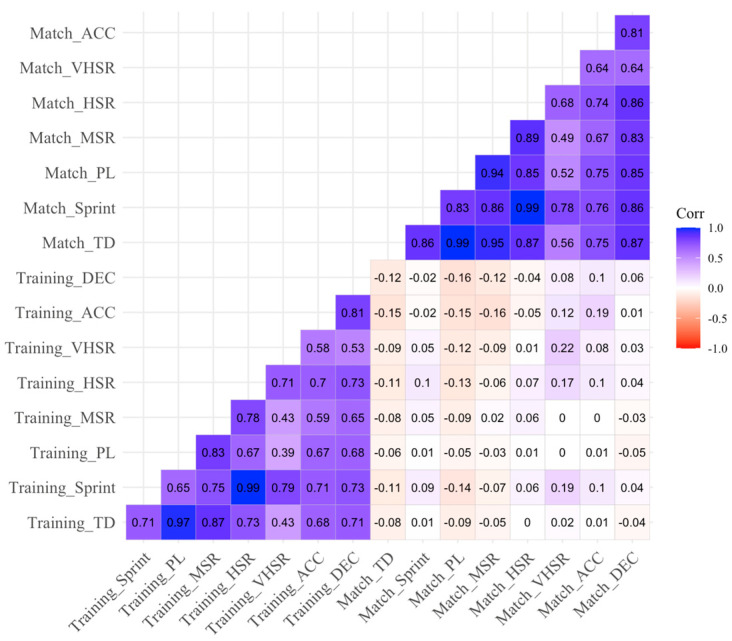
Relationships between accumulated TL and match physical demands. Note. TD = total distance; MSR = medium-speed running; HSR = high-speed running; VHSR = very high-speed running; Sprint = sprint-speed running distance; ACC = number of accelerations; DEC = number of decelerations; PL = player load.

Figure 1 depicts the correlations between weekly accumulated external load and match values of the same week for each variable. Trivial and negative correlation coefficients were found between weekly external load and match demands for TD (*r* = −0.08) and PL (*r* = −0.05); trivial and positive correlations were found for MSR (*r* = 0.02), HSR (*r* = 0.07), Sprint (*r* = 0.09), and DEC (*r* = 0.06); and small and positive correlations were found for VHSR (*r* = 0.22) and ACC (*r* = 0.19).

### 3.3. Training and Match External Load Ratios

Table 2 present the descriptive statistics (means ± standard deviations) of daily TMr, according to the training sessions. The greatest TDr, MSRr, VHSRr, Sprintr, HSRr, and PLr occurred in MD–3. However, the highest ACCr and DECr occurred in MD–4. Contrarily, the lowest TDr, MSRr, VHSRr, HSRr, ACCr, DECr, and PLr appeared in MD–2. The lowest Sprintr occurred during MD–1.

## 4. Discussion

The aim of this study was twofold: (i) to analyze the relationship between weekly accumulated TL and match running performance in the same week and (ii) to describe the training/match ratios of different external load measures considering variations across different training days. This study provides new insights about the relationship between weekly accumulated TL and match physical demands since previously conducted studies [18,26]. The main results showed that (i) trivial-to-small correlations were observed between the weekly TL and the match physical demands of that week for different external load variables; (ii) the greatest values of TD, MSR, VHSR, Sprint, HSR, and PL occurred during MD–3, and the highest values of ACC and DEC occurred during MD–4, showing that the hardest training sessions were those days; and (iii) the TMr confirmed the same physical demands for the same training sessions, where the external load variables were the greatest.

Prior studies have informed about the influence of weekly TL on match physical demands in soccer competition, showing that decreasing TL was associated with an increase in physical activities during matches [18]. Our main results reported trivial-to-small correlations between the weekly accumulated TL and match physical demands. Concretely, trivial and negative correlations were found for TD and PL. Although these relationships are poor, the results showed the importance of preparing for competition. For example, when soccer players performed less than weekly TL, their match running performance from the same week was significantly higher [18], possibly explained by the better player recovery during the week, ensuring a better readiness for the competition. Contrarily, small and positive correlations were found for VHSR and ACC, which shows it would be beneficial for soccer athletes to be exposed to VHSR and ACC demands similar to those observed during matches [21]. Our results also reported similar relationships to previous studies between the prior weekly training volume and subsequent relative HSR and ACC match physical demands [35]. Nevertheless, the magnitude of these correlations suggest that soccer weekly TL is independent of subsequent match loads, due to the nature of the match (e.g., contextual-related variables) and possibly also due to variations in the time available to train for the next match (i.e., different number of sessions; [20]).

The weekly TL distribution has been exhaustively analyzed in professional soccer, mainly considering the number of training sessions remaining before the match and the days passed since the last match played [14,24]. The day after the match (i.e., MD+1) is usually used for recovery training sessions for players who have played more minutes [36], while players who have played less perform a top-up session. Our results showed low values for different external load variables indicating the low volume and intensity of this type of training session. On central days of the microcycle (i.e., MD–4 and MD–3), higher loads are accumulated due to the greater distance from the competition [37], aiming for players to achieve physical readiness to the demands of competition. In both MD–3 and MD–4, the greatest values for all physical variables occurred, showing that the external load in training sessions were closest (or even higher) to the match physical demands during those training days. Finally, external load measures were decreased on days close to the next match (i.e., MD–2 and MD–1) to ensure players’ readiness and optimal performance for competition [19].

The results reported support those obtained in previous studies, which have analyzed the weekly TL distribution in different professional soccer teams from the English Premier League [38,39], Dutch Eredivisie [27], First Portuguese leagues [20], and Spanish leagues [40,41]. This type of study is important, as cultural and competition demands between different soccer leagues could provide different TL distributions in order to optimize the players’ performance. However, similarly to our results, these studies showed the lowest TL on MD–1 and the highest TL on MD–3 and MD–4. For example, the greatest ACCr and DECr occurred on MD–3, where such training structure is typical of sessions with small-sided games, the highest MSRr, HSRr, VHSRr, and Sprintr occurred in MD–4 with large-sided games [42,43], and the lowest TMr occurred in MD–2 and MD–1, focusing on the tapering for next match [44]. This distribution could be due to, within weeks, avoiding excessive training loads on the days closest to the match, which allows for better performance during the match [44]. In addition, the highest TMr for high-intensity distance variables should be reached at MD–4, as the results reported, due to recent studies, have informed that a 72 h recovery period may not be adequate for hamstring muscles in terms of hamstring injury risk factors [45]. Therefore, TL distribution must consider several factors and variables when planning the TL over different weeks and even vary within the days of the week, as all of these elements can influence match performance [19,24].

### Limitations and Future Directions

It is relevant to highlight some issues of the present research to be addressed in further studies. The main limitations were related to the small sample size and the fact that only one team was involved, which makes it difficult to generalize the results. This is a very common issue among studies conducted in professional soccer players [40,46]. Also, only players who participated in full training sessions and the corresponding following matches were included in the analysis, so the sample was reduced. In addition, any internal load variables (i.e., heart rate, subjective perceived exertion or subjective feelings of mental load) were not included in the research. Further studies should include these measures to obtain a holistic and more comprehensive approach of TL. On the other hand, the analysis was conducted with all the sample size without considering the player participation in soccer matches, so the use of groups depending on the minutes played in the previous match could provide new and practical information in future studies. In addition, further studies should introduce the relationship between daily TL and match physical demands in order to describe the influence of external load day-by-day on subsequent matches and obtain a better and more precise management on TL during daily training sessions. Another important issue is whether higher weekly TL led to better match performance (i.e., win matches), so next studies could include the causal analysis between TL and match performance. Finally, these results found a weak correlation between TL and match physical demands, so it should be recommended to further explore other potential factors influencing match physical demands, such as individual differences (e.g., different playing positions may have varying physical demands during matches), tactical requirements, or psychological factors.

## 5. Conclusions

Firstly, the data demonstrated that, although the magnitude of the correlations suggest that soccer weekly TL is independent of subsequent match loads, trivial and negative correlations were found for TD and PL between weekly TL and match physical demands, and small and positive correlations for VHSR and ACC. These findings suggest that avoiding highly accumulated TL during microcycles would increase the TD covered by soccer players in official matches. Secondly, the TMr showed that MD–3 and MD–4 were the hardest training sessions. A correct weekly TL distribution could optimize the subsequent performance of soccer players. The present study highlights the need for coaches and technical staff to consider the importance on planning and TL monitoring in order to optimize physical performance and prevent injuries.

### Practical Applications

These results may provide useful knowledge for soccer coaches and strength and conditioning staff to better understand the distribution of weekly TL and their influence on match physical demands. Specifically, our results would make it easier to determine how to manage TMr. Some practical applications include (i) players should be exposed to a minimum of 80% of the intensity related to match intensity in the middle of the week (i.e., on MD–4 MD–3 in our study); (ii) running-based drills should be necessary during training microcycles to increase the match running performance of soccer players in terms of high-intensity actions; (iii) practitioners should reduce the volume of the training sessions after heavy training sessions characterized by high external load in MD–4 and MD–3; (iv) managing which types of ratios improve performance and reduce the risk of injury; and (v) MD+1 sessions could be the most important sessions to compensate the competitive stimulus for soccer players who did not participate in previous matches.

## Figures and Tables

**Table 1:** Daily and match external load measures according to the type of training day.

Variables	MD+1	MD–4	MD–3	MD–2	MD–1	MD	Total
	Coeff (SE)	Coeff (SE)	Coeff (SE)	Coeff (SE)	Coeff (SE)	Coeff (SE)	Coeff (SE)
TD (m)	4322 (114)	6677 (105)	7316 (107)	3661 (110)	3938 (113)	10,587 (119)	34,561 (3182)
MSR (m)	710 (54.7)	1459 (52.7)	1715 (53.1)	490 (53.7)	482 (54.4)	2945 (55.9)	7252 (1391)
VHSR (m)	166 (17.7)	310 (17.1)	442 (17.2)	103 (17.4)	110 (17.6)	806 (18.1)	1781 (445)
Sprint (m)	15.22 (4.22)	27.09 (4.07)	55.22 (4.10)	7.57 (4.15)	3.08 (4.20)	124.31 (4.31)	206 (104)
HSR (m)	181 (20.8)	337 (20.1)	498 (20.3)	110 (20.5)	113 (20.7)	930 (21.3)	1987 (524)
ACC (n°)	14.4 (1.55)	25.7 (1.53)	20.5 (1.53)	13.7 (1.54)	15.1 (1.55)	33.0 (1.56)	114 (32.92)
DEC (n°)	12.14 (1.11)	27.31 (1.08)	21.80 (1.09)	9.59 (1.10)	10.30 (1.11)	40.75 (1.33)	112 (27.82)
PL (m)	227 (6.04)	325 (5.72)	338 (5.79)	197 (5.88)	216 (6.00)	478 (6.24)	1697 (164)

Note. Coeff = coefficient; SE = standard error; m = meters; TD = total distance; MSR = medium-speed running; HSR = high-speed running; VHSR = very high-speed running; Sprint = sprint-speed running distance; ACC = number of accelerations; DEC = number of decelerations; PL = player load; MD+1 = one day following the match; MD–4 = four days before the match; MD–3 = three days before the match; MD–2 = two days before the match; MD–1 = one day before the match; and MD = match day.

**Table 2:** Training/match external load ratios based on the type of training day.

TMr	MD+1	MD–4	MD–3	MD–2	MD–1
	M (SD)	M (SD)	M (SD)	M (SD)	M (SD)
TDr	0.70 (1.08)	1.08 (1.69)	1.19 (1.92)	0.06 (0.94)	0.07 (1.12)
MSRr	4.19 (34.27)	9.04 (77.73)	10.46 (88.84)	3.84 (35.66)	4.13 (34.92)
VHSRr	1.32 (14.70)	2.26 (24.97)	3.25 (36.60)	0.72 (7.93)	0.79 (9.06)
Sprintr	0.27 (0.70)	0.43 (1.32)	0.81 (2.48)	0.12 (0.33)	0.04 (0.20)
HSRr	1.42 (16.30)	2.38 (27.18)	3.55 (41.82)	0.75 (8.62)	0.79 (9.24)
ACCr	0.62 (0.82)	1.06 (1.19)	0.82 (0.91)	0.55 (0.65)	0.60 (0.70)
DECr	0.58 (1.25)	1.18 (2.29)	0.93 (1.76)	0.40 (78)	0.42 (0.73)
PLr	0.71 (82)	1.02 (1.13)	1.07 (1.21)	0.61 (0.68)	0.69 (0.83)

Note. M = mean values; SD = standard deviation; TMr = training/match ratios; TDr = total distance ratio; MSRr = medium-speed running ratio; HSRr = high-speed running ratio; VHSRr = very high-speed running ratio; Sprint = sprint-speed running distance ratio; ACCr = accelerations ratio; DECr = decelerations ratio; PLr = player load ratio; MD+1 = one day following the match; MD–4 = four days before the match; MD–3 = three days before the match; MD–2 = two days before the match; MD–1 = one day before the match; and MD = match day.

## Data Availability

The data are available upon request to the corresponding author.

## Abbreviations

The following abbreviations are used in this manuscript:

TL	Training load
TD	Total distance
MSR	Medium-speed running
HSR	High-speed running
VHSR	Very high-speed running
Sprint	Sprinting-speed running
ACC	Accelerations
DEC	Decelerations
MD	Match day
TMr	Training/match ratio
